# Unstable metaphors, uncertain minds: how metaphors shape judgments and opinions

**DOI:** 10.3389/fpsyg.2025.1536950

**Published:** 2025-04-01

**Authors:** Martina Montalti, Stefana Garello, Valentina Cuccio

**Affiliations:** ^1^Department of Ancient and Modern Civilizations, University of Messina, Messina, Italy; ^2^Unit of Neuroscience, Department of Medicine and Surgery, University of Parma, Parma, Italy; ^3^Department of Philosophy, Communication and Arts, University of Roma Tre, Rome, Italy

**Keywords:** conceptual metaphors, physical instability, embodied cognition, embodied persuasion, metaphor processing, embodied language

## Abstract

**Introduction:**

Previous research has shown that experimentally manipulated physical states of instability negatively affect judgments and opinions. Based on this result, in the current study we want to investigate whether the processing of metaphorical expressions related to physical instability (e.g., “Our economy is shaky”) and underpinned by the conceptual metaphor EMOTIONAL STABILITY IS BALANCE negatively affects participants’ judgments.

**Methods:**

Three hundred participants were assigned to three experimental groups. Each group was presented with sentences containing, respectively, stable metaphors, unstable metaphors or unstable literal sentences. After reading the sentences, participants were asked to respond to six topical questions regarding current and future economic and life perspectives.

**Results:**

Data shows that across all six topical questions, reading stable metaphors led to more positive judgments compared to unstable sentences (both metaphors and literal ones). Moreover, in four questions out of six, reading unstable metaphorical sentences led to lower ratings compared to unstable literal sentences.

**Discussion:**

These findings support the main claim of Conceptual Metaphor Theory, i.e., the idea that abstract and complex concepts are structured via metaphorical mappings from more concrete and simple ones and align with the Embodied Cognition Hypothesis. Furthermore, these findings suggest that metaphors can have a powerful influence on our perception of the world and contribute to our understanding of how metaphorical language can shape bodily experiences and attitudes.

## 1 Introduction

Empirical studies have investigated the relationship between physical stability or instability and the abstract concepts of certainty and uncertainty (e.g., Kille et al., 2013; Landau et al., 2010; Williams, 2024). These studies show that physical states of stability and instability influence people’s judgments and opinions in different ways. To investigate the psychological significance of this link, Kille et al. (2013) conducted an experiment in which participants were assigned to either a stable or an unstable workstation. They were then asked to evaluate the relationships of public figures (e.g., “Barack and Michelle Obama”). The results showed that participants in the unstable workplace viewed relationships as less stable than those in the stable workplace. In addition, participants in the unstable workplace placed less emphasis on characteristics associated with stability, such as “loyalty,” when envisioning their ideal partner.

This pattern was confirmed by Forest et al. (2015), who conducted a similar study. In their study, participants who were in physically unstable scenarios—such as balancing on one leg or sitting on an inflatable pillow—expressed less affection for their partner and rated their romantic relationships more negatively than participants in stable situations. These results suggest that physical instability can have an impact on how people perceive their relationships.

Li (2021) expanded on this topic by examining the relationship between physical instability and mental instability in relation to coronavirus anxiety. In three experiments, they found that physical instability increased participants’ anxiety about COVID-19. In the first experiment, participants filled out a survey while sitting on a wobbly desk and chair, and they reported greater anxiety about COVID-19 than those sitting in a stable environment. The second experiment showed that participants who stood on one-foot experienced higher levels of anxiety than those who stood on both feet. In the third experiment, the researchers examined subsequent behaviors related to COVID-19 attitudes. Participants exposed to unstable conditions donated more money to a program focused on developing new COVID-19 treatments than participants in stable conditions. These findings suggest that physical instability can affect people’s perceptions and behaviors about their health and relationships.

Overall, these results suggest that our understanding of abstract concepts (e.g., emotional instability) evolves in parallel with our physical experiences (e.g., physical instability) (Bolognesi and Steen, 2018; Borghi and Binkofski, 2014; Cuccio, 2022; Villani et al., 2022). In other words, a physically unstable state can have a negative impact on people’s evaluations and opinions, while physical stability can promote a more positive attitude toward the same topic.

These findings are consistent with the core ideas of Conceptual Metaphor Theory (CMT; Lakoff and Johnson, 1980; Lakoff, 1987, 1993), which assumes that abstract and complex domains in human cognition are organized by metaphorical mappings derived from more concrete and physical experiences. For example, we understand “time” as “money” when we say, “spending time.” These mappings are systematic and grounded in sensory-motor experiences, like associating “up” with positive emotions (e.g., “feeling high”) and “down” with negativity (“feeling low”). CMT suggests that metaphors are not just linguistic but fundamental to human thought, shaping how we reason and make sense of the world.

Furthermore, CMT is consistent with the Embodied Cognition Hypothesis, which states that cognitive processes are essentially based on sensory-motor experiences. These experiences allow individuals to acquire knowledge and build conceptual frameworks based on their physical interactions with the environment (Cuccio, 2022; Gallagher, 2005; Gallese and Lakoff, 2005; Gibbs, 2005; Montalti et al., 2024). In this respect, a key aspect of human cognition is neural exploitation, “the adaptation of sensory-motor brain mechanisms to serve new roles in reason and language, while retaining their original functions as well” (Gallese and Lakoff, 2005, 2). For instance, the same parietal-premotor neural circuits that are active when we grasp something are also involved in structuring the concept of “grasping” and in processing sentences that contain the verb “grasp,” such as “I grasped the glass” (Buccino et al., 2005; Raposo et al., 2009; Bedny et al., 2011; Mirabella et al., 2012; Fernandino et al., 2013). The same mechanism, known as Embodied Simulation (Gallese and Sinigaglia, 2011), underlies also the comprehension of abstract and metaphorical concepts: it seems that even the sentence “I grasped the idea” activates the same brain regions that are active in the action of grasping something (Gibbs, 2006; Cacciari et al., 2011; Cacciari and Pesciarelli, 2013; Desai et al., 2011, 2013; Romero Lauro et al., 2013; Garello et al., 2024). These results suggest that there is no gap between perception, action, cognition and language, but rather that they are rooted in the perception-action circuit. From this perspective, abstract concepts such as certainty and uncertainty are characterized by metaphorical connections to physical experiences of stability and instability. Building on the results of previous already mentioned research in which experimenters manipulated physical states of stability and instability and their relation with people’s moral judgments and attitudes (Li, 2021; Kille et al., 2013; Landau et al., 2010; Williams, 2024), the present study hypothesizes that similar effects on participants’ judgments and opinions can be elicited by processing metaphors related to physical stability and instability.

These metaphorical expressions are widely used in many languages. Specifically, a notable link exists between the physical realm of instability and abstract concepts such as moral uncertainty and mental imbalance. Common metaphorical expressions such as “my relationship is unstable,” “we are in a shaky situation,” “I have lost my psychophysical balance” or “I have been stuck in a rut for many years” appear frequently in everyday language. These expressions are by no means arbitrary but seem to reflect a consistent psychological and embodied link between physical instability and mental instability.

In particular, these expressions seem to reflect the conceptual metaphor EMOTIONAL STABILITY IS BALANCE, that frames emotional well-being with physical balance, suggesting that just as maintaining physical balance requires steadiness and equilibrium, emotional stability involves maintaining a calm and centered psychological state (cf. *Master Metaphor List*, Lakoff et al., 1991). When someone is emotionally balanced, they are metaphorically stable and not affected by emotions or external events. In contrast, emotional turbulence is often expressed as “losing balance,” “being unstable” or “being in a shaky situation.” The conceptual metaphor EMOTIONAL STABILITY IS BALANCE encompasses both the aspect of emotional stability and emotional instability and is rooted in our embodied experience of physical balance/imbalance, which can be easily understood and applied to emotional states.

Building on these theoretical and empirical findings, we hypothesize that processing a list of sentences composed by unstable metaphors (e.g., “this relationship is unstable,” “our economy is shaky,” “the result is teetering”) will negatively affect the judgments of participants on topics such as the current economic situation, the development of the COVID-19 pandemic, the future perspectives, the job market, and the common social problems in Europe, compared to the processing of a list of sentences composed of stable metaphors. If our prediction is confirmed, the findings will not only align with the embodiment of language framework but also reinforce the role of the body in persuasion, i.e., the process through which bodily experiences, physical actions and sensory perceptions can influence belief processes and the changing of opinions or attitudes (Petty and Brinol, 2008; Cuccio, 2016). They will demonstrate how metaphorical expressions tied to physical instability can shape evaluations and attitudes, extending and reinforcing our understanding of embodied cognition and its influence on abstract reasoning.

## 2 Materials and methods

### 2.1 Participants

The required sample size was estimated using G*Power (version 3.1.9.4; Faul et al., 2009) for a one-way fixed effects ANOVA (effect size = 0.20, power = 0.85, a Type I Error Rate = 0.05, and three experimental groups), resulting in a minimum sample of 279. To meet this requirement, 300 volunteers (50% female) without any psychiatric or neurological disorder were recruited by posting announcements on social networks, and word of mouth. All participants were native Italian speakers and were not informed about the study’s purpose. Participants were randomly assigned to one of three experimental groups. Groups were equally sized (100 participants in each), gender-balanced (50 females, 50 males), and did not differ in terms of both age, as assessed by a one-way Kruskal-Wallis rank sum test (H(2) = 1.33, *p* = 0.514), and education as assessed by a Chi-Square Test for Homogeneity [*χ^2^*(6) = 11.34; *p* = 0.078]. See Table 1 for more information about participant’s characteristics.

**Table 1:** Demographic information of the three experimental groups.

	Group 1 Metaphorical stable	Group 2 Metaphorical unstable	Group 3 Literal unstable
Gender (F/M)	50/50	50/50	50/50
Age (M ± SD) [range]	37.2 ± 13.1 [18–76]	36.6 ± 12.1 [22–71]	35.9 ± 13.1 [19–74]
Education			
Middle school diploma	20	19	16
High school diploma	53	56	39
Degree	26	24	43
PhD or master	1	1	2

Data were collected between May and September 2021. Due to the COVID-19 pandemic, the study was conducted entirely online using Google Forms. The study was approved by the local ethical committee (Comitato Etico, COSPECS Department, University of Messina; approval number given: COSPECS_3_2021) and was conducted in accordance with the World Medical Association (2013). All participants were informed about the task and provided informed consent to participate by selecting “I consent to participate in this study.” In such a way, participants declare that they voluntarily adhere to the implementation of the research, and that they are aware that the recorded data will be analyzed only for research purposes in absolute anonymity.

### 2.2 Stimuli

The stimuli were designed based on the Master Metaphor List (Lakoff et al., 1991), a comprehensive collection of over 791 carefully curated conceptual mappings, designed to serve as a definitive research tool for metaphor studies. Specifically, we focused on the conceptual metaphor EMOTIONAL STABILITY IS BALANCE, which encompasses both stability and instability as manifestations of the same conceptual domain. Examples of related metaphorical expressions include: “He is unbalanced,” “She’s not on an even keel,” “They’re upset,” “I’m a very stable individual” and “She’s quite level-headed” (cf. Lakoff et al., 1991, 150). Following this framework, we developed three different lists of Italian sentences: (1) 13 metaphorical expressions referring to a state of physical stability, (2) 13 metaphorical expressions related to a physically unstable condition, and (3) 13 literal expressions describing a physically unstable condition (see Table 2). Particular attention was given to the condition of physical instability within this conceptual metaphor. This focus allowed us to explore the nuanced representations of instability as a central aspect of the metaphorical mapping. At the same time, we included stable metaphorical and unstable literal conditions as controls. Specifically, stable metaphors served as a control for the condition of physical balance (we compared stable vs. unstable physical condition). Given that stable metaphors typically carry a positive valence, while unstable metaphors tend to have a negative valence, we sought to rule out the possibility that the observed effects might be driven by the valence of the stimuli rather than their metaphorical nature. To address this, we included a literal unstable condition, providing both a semantic and valence control. Since the literal unstable sentences have negative valence, this approach allowed us to ensure that any observed effects could be specifically attributed to the metaphorical nature of the expressions, rather than merely the valence of the stimuli.

**Table 2:** Examples of linguistic stimuli and descriptive statistics for sentence length and frequency of use.

Metaphorical stable	Metaphorical unstable	Literal unstable
Il nostro sentimento è **saldo** *Our sentiment is firm*	La nostra economia è **traballante** *Our economy is unstaedy*	Il mio dente è **traballante** *My tooth is unsteady*
La situazione è **statica** *The situation is static*	Questo matrimonio è in **bilico** *This marriage is* teetering	Questo peso è **sbilanciato** *This weight is unbalanced*
La tua posizione è **inamovibile** *Your position is unmovable*	Il risultato è in **bilico** *The result is teetering*	Questo ponte è **pericolante** *This bridge is rickety*
**Number of characters (M ± SD)**		
27.1 ± 3.3	27.1 ± 3.4	24.7 ± 3.7
**Number of syllables (M ± SD)**		
10.1 ± 1.8	10.1 ± 1.3	8.7 ± 1.2
**Number of words (M ± SD)**		
4.2 ± 0.8	4.5 ± 0.7	4.2 ± 0.8

The sentences were taken from the Italian Web Corpus (ItTenTen 2020), an Italian corpus consisting of texts gathered from the Internet, containing a total of 12.4 billion words.

We first ensured that the three sentence lists were balanced in terms of lenght. As Shapiro–Wilk tests revealed that not all variables were normally distributed, we employed parametric ANOVA or non-parametric Kruskal Wallis test [Sentence (3 levels, Unstable Metaphors, Stable Metaphors, and Unstable Literals)]. The three lists of sentences were balanced for number of characters [parametric ANOVA; *F*(2,36) = 2.11; *p* = 0.14; partial eta square (*η*^2^_p_) = 0.11], syllables [Kruskal-Wallis rank sum test; H(2) = 6.95, *p* = 0.031, eta square (*η*^2^) = 0.14, although none of the post-hoc comparisons remained significant after Benjamini-Hochberg correction; all *p_s_* > 0.05], and words [Kruskal-Wallis rank sum test; H(2) = 3.49, *p* = 0.175, *η*^2^ = 0.04]. Additionally, we included 13 neutral literal sentences as filler items in each list to decrease the homogeneity of the stimuli. Thus, each of the three lists of sentences was composed of 26 sentences.

### 2.3 Experimental design and procedure

We created three questionnaires with the same structure and duration. Each questionnaire was constituted of three sections. In the first one, we collected demographic information, i.e., age, gender, and education. In the second one, the 26 sentences were presented, and participants were asked to read and evaluate the valence and familiarity of each sentence. Valence was assessed using a Likert scale that ranged from 1 (negative) through 5 (neutral) to 10 (positive). Familiarity was also assessed on a Likert scale ranging from 1 (not at all familiar) to 10 (very familiar). Questions were administered with two-fold aims. First, to ensure that participants were reading and processing the sentences. Second, to control valence and familiarity of the stimuli. In the third section of the questionnaire, participants were asked to express a judgment about six topical arguments. Specifically, topical questions were related to the current Italian health situation (Q1: “How do you evaluate the current Italian health care system?”; Q2: “How do you assess the measures taken in Italy to deal with the COVID-19 pandemic?”), the Italian economic situation in the near future (Q3: “How do you imagine the Italian economic situation in the coming years?”), the future social life (Q4: “How do you imagine social life in the coming years?”), and the current educational and future work situations (Q5: “How do you evaluate smart working and distance learning?”; Q6: “How do you imagine your work situation in the coming years?”). Participants responded using a Likert scale ranging from 1 (extremely negative) to 10 (extremely positive).

### 2.4 Data analysis

Separately for each experimental question, we performed a one-way ANOVA [between-participants factor: Sentence (three levels: unstable metaphors, stable metaphors, unstable literal sentences)]. Since all variables were not normally distributed, as assessed by the Shapiro–Wilk test, we used a series of non-parametric Kruskal-Wallis tests. We also analyzed valence and familiarity ratings using a one-way ANOVA [between-participants factor: Sentence (three levels: unstable metaphors, stable metaphors, unstable literal sentences)]. All post-hoc tests were performed using Benjamini-Hochberg corrections, while the effect sizes were quantified as eta-squared (*η*^2^). For each test, we computed Bayes Factors (BF_10_; Jarosz and Wiley, 2014) using prior odds of 0.707 to quantify the null hypothesis’ strength (R package BayesFactor; Morey et al., 2024). BF_10_ provide a ratio of evidence for two competing models, i.e., H1 vs. H0, quantifying the strength of evidence in favor of one hypothesis over the other. BF_10_ values >3 and > 10 indicate moderate and strong support for the alternative hypothesis, respectively. BF_10_ Values <0.1 and < 0.33 provide strong and substantial evidence for the null hypothesis, while BF_10_ values between 0.33 and 3 are considered inconsistent for any hypothesis. All statistical analyses were conducted using R version 4.2.3 (R Core Team, 2020). The datasets analyzed during the current study are available in the OSF repository, https://osf.io/8wgae/?view_only=7ce40895aac44b86a978509ce8a54984.

## 3 Results

**Figure 1:**
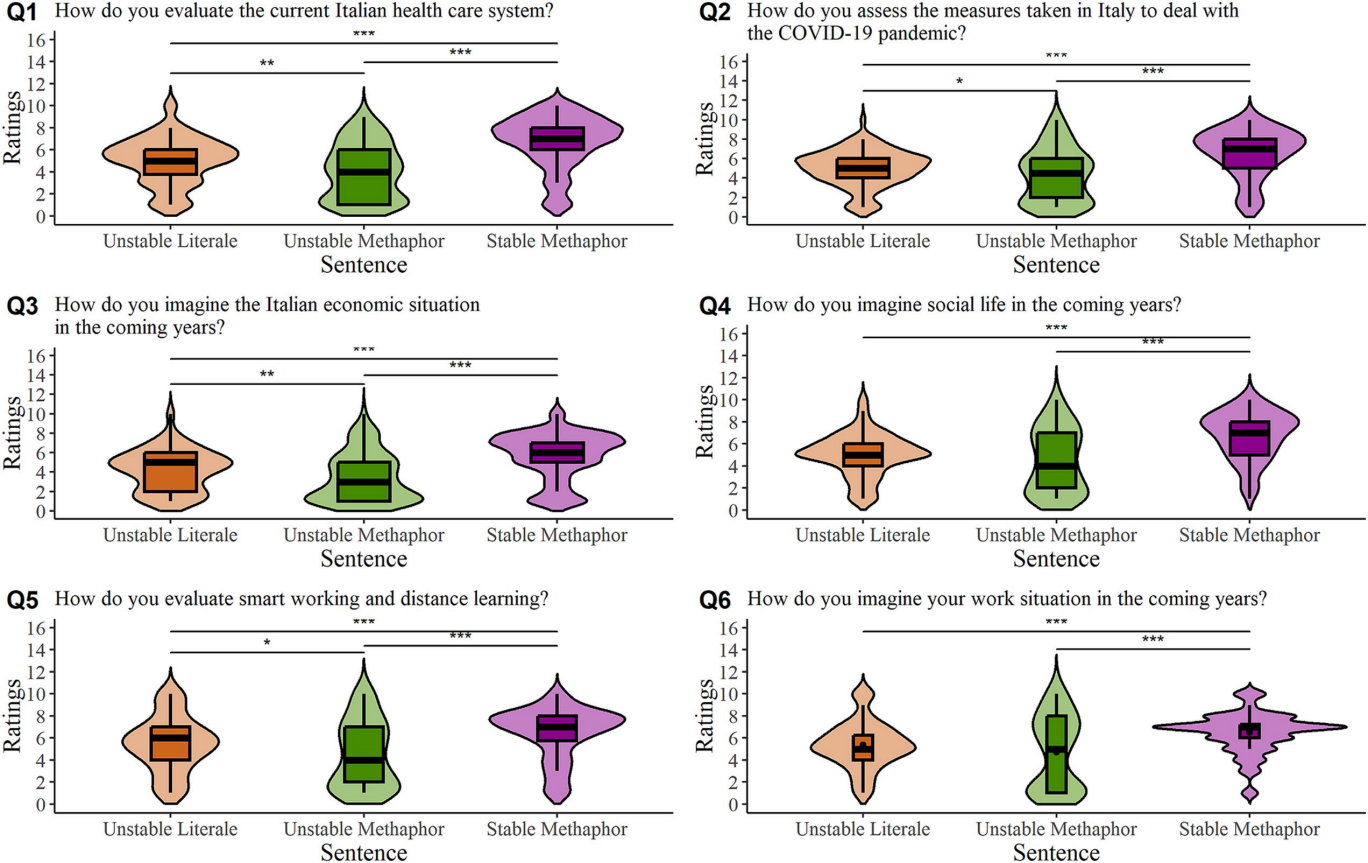
Violin plots of participant ratings of the six topical questions (each panel corresponds to one question – Q1-Q6). The black horizontal line indicates the median, the box plot’s lower and upper edges represent the 1st and 3rd quartiles, and the density curve of the violin graph indicates the frequency of observations. * = *p* < 0.05; ** = *p* < 0.01; *** = *p* < 0.001.

The Kruskal-Wallis test applied to the first question (Figure 1, panel Q1) was significant [H(2) = 58.20, *p* < 0.0001; *η*^2^ = 0.19; BF_10_ > 100]. *Post-hoc* comparison showed that all three groups of participants differed significantly from each other, since participants that read metaphorical unstable sentences (M ± SD = 4.0 ± 2.5) gave significantly lower ratings compared to those that evaluated both literal unstable sentences (M ± SD = 5.0 ± 2.2; *p* < 0.001; BF_10_ > 10) and metaphorical stable sentences (M ± SD = 6.7 ± 2.4; *p* < 0.0001; BF_10_ > 100). Furthermore, those who read literal unstable sentences responded significantly more negatively to Q1 than those who read the metaphorical stable ones (*p* < 0.0001; BF_10_ > 100).

The analysis performed on the second question (Figure 1, panel Q2) showed the same pattern, with a significant difference between all conditions [H(2) = 46.36, *p* < 0.0001; *η*^2^ = 0.15; BF_10_ > 100]. Specifically, post-hoc comparison demonstrated that those who read the metaphorical unstable sentences (M ± SD = 4.3 ± 2.7) gave significantly lower ratings compared to those who read the literal unstable sentences (M ± SD = 5.0 ± 1.9; *p* < 0.05; BF_10_ = 1.14) and the metaphorical stable sentences (M ± SD = 6.6 ± 2.4; *p* < 0.0001; BF_10_ > 100). As before, participants who read the literal unstable sentences gave significantly more negative responses compared to those who read the metaphorical stable ones (*p* < 0.0001; BF_10_ > 100).

The same pattern was found in question three (Figure 1, panel Q3), as indicated by a significant Kruskal-Wallis test [H(2) = 42.34, *p* < 0.0001; *η*^2^ = 0.14; BF_10_ > 100] and post-hoc comparison. All groups differed significantly from each other, since participants that read metaphorical unstable sentences (M ± SD = 3.5 ± 2.5) gave significantly lower ratings compared to both those that read literal unstable sentences (M ± SD = 4.2 ± 2.1; *p* < 0.01; BF_10_ = 1.87) and those that read metaphorical stable ones (M ± SD = 5.7 ± 2.4; *p* < 0.0001 BF_10_ > 100). Moreover, participants who read literal unstable sentences gave more negative responses compared to those who read the metaphorical stable ones (*p* < 0.0001 BF_10_ > 100).

As for question four (Figure 1, panel Q4), the Kruskal-Wallis test was significant [H(2) = 44.79, *p* < 0.0001; *η*^2^ = 0.14; BF_10_ > 100]. A post-hoc comparison revealed that the participants who read the metaphorical stable sentences (M ± SD = 6.8 ± 2.2) had significantly higher ratings than both the participants who read the metaphorical unstable sentences (M ± SD = 4.5 ± 2.8; *p* < 0.0001; BF_10_ > 100) and those who read the literal unstable sentences (M ± SD = 5.0 ± 1.9; *p* < 0.0001; BF_10_ > 100). However, in this case, there was no significant difference between the metaphorical unstable and literal unstable sentences (*p* = 0.16; BF_10_ = 0.45).

In the fifth question (Figure 1, panel Q5), the Kruskal-Wallis test was significant [H(2) = 27.18, *p* < 0.0001; *η*^2^ = 0.085; BF_10_ > 100] and post-hoc comparison showed that all three groups of participants differed significantly from each other, since participants who read the metaphorical unstable sentences (M ± SD = 4.5 ± 3.0) gave significantly lower ratings than both participants who read the literal unstable sentences e (M ± SD = 5.4 ± 2.5; *p* = 0.02; BF_10_ = 2.48) and those who read the metaphorical stable sentences (M ± SD = 6.4 ± 2.3; *p* < 0.0001; BF_10_ > 100). Also in this case, participants who read the literal unstable sentences gave significantly more negative ratings than those who read the metaphorical stable ones (*p* < 0.0001; BF_10_ = 8.20).

Lastly, also in question six (Figure 1, panel Q6) the Kruskal-Wallis test was significant [H(2) = 22.78, *p* < 0.0001; *η*^2^ = 0.070; BF_10_ > 100]. As in question four, the post-hoc comparison showed that the group who read the metaphorical stable sentences (M ± SD = 6.5 ± 1.8) gave significantly higher responses compared to both the group who read the metaphorical unstable sentences (M ± SD = 4.7 ± 3.3; *p* < 0.001; BF_10_ > 100) and the literal unstable sentences (M ± SD = 5.4 ± 2.1; *p* < 0.0001; BF_10_ > 100) groups. The unstable metaphors group and the unstable literal sentences group did not differ (*p* = 0.25; BF_10_ = 0.57).

### 3.1 Valence and familiarity

The Kruskal-Wallis test on valence ratings showed a significant main effect of Sentence [H(2) =136.31, *p* < 0.0001; *η*^2^ = 0.45]. As expected, post-hoc comparison revealed that the group who evaluated the metaphorical stable sentences (M ± SD = 8.2 ± 1.7) gave significantly higher ratings compared to both the groups who read the metaphorical unstable sentences (M ± SD = 3.8 ± 2.5; *p* < 0.0001) and the literal unstable sentences (M ± SD = 3.4 ± 2.8; *p* < 0.0001). No significant difference was found between the metaphorical unstable and literal unstable sentence groups (*p* = 0.06).

The Kruskal-Wallis test on familiarity ratings was also significant [H(2) = 19.73, *p* < 0.0001; *η*^2^ = 0.06]. Post-hoc comparison indicated that stable metaphorical sentences (M ± SD = 8.5 ± 1.8) were perceived as more familiar than both unstable metaphorical (M ± SD = 7.1 ± 2.3; *p* < 0.0001) and unstable literal (M ± SD = 7.6 ± 2.2; *p* = 0.001) sentences. No significant difference was found between the two types of unstable sentences (*p* = 0.16).

## 4 Discussion

In the present study, we investigated whether unstable metaphorical expressions, underpinned by the conceptual metaphor EMOTIONAL STABILITY IS BALANCE, negatively influence participants’ judgments on sensitive topics such as the current economic situation, the progression of the pandemic, future prospects, the job market, and the common social problems in Europe.

Specifically, we found that in all the six topical questions the exposure to unstable metaphors led to more negative judgments toward the target concepts whereas stable metaphors have a positive effect leading to higher ratings. In addition to this, and interestingly, in four questions out of six (Q1, Q2, Q3, Q5), reading unstable metaphorical sentences also led to lower ratings compared to the ratings produced by the group of participants who read the literal unstable sentences. With regard to valence and familiarity of the sentences used in the study, we found that stable metaphors significantly differ from both unstable metaphors and unstable literal sentences. Indeed, stable metaphors are more positively valenced (as it was easily expected) and more familiar than both unstable metaphors and unstable literal sentences.

These findings align with previous research on the relationship between physical stability and abstract concepts like certainty and uncertainty (e.g., Kille et al., 2013; Landau et al., 2010; Williams, 2024) according to which physical instability negatively affects participants’ moral judgments and attitudes, influencing also their behaviors and highlighting the broader psychological implications of instability, showing how sensory-motor experiences can shape evaluations, attitudes, and behaviors across different contexts. By comparing our findings with this body of work, we suggest that metaphorical instability, as reflected in language, mirrors the effects observed in physical instability. This strengthens the argument for an embodied cognition framework, where linguistic metaphors tied to sensory-motor schemas can systematically influence abstract reasoning and decision-making.

On the other hand, and interestingly, unstable metaphors and unstable literal sentences do not differ from each other in terms of both valence and familiarity. This last result is particularly interesting for two reasons. Firstly, considering that both unstable metaphors and unstable literal sentences have the same valence and the same familiarity but in four questions out of six (Q1, Q2, Q3, Q5), reading unstable metaphorical sentences led to lower ratings compared to the ratings produced by the group of participants who read the literal unstable sentences, we can rule out the possibility that lower ratings were determined exclusively or predominantly by the valence or familiarity of the sentences. Secondly, the fact that the processing of unstable metaphors negatively affected the judgments of the participants significantly more compared to the experimental condition in which participants read literal unstable sentences seems to suggest that the activation of the conceptual metaphor which links together physical instability (“source” or “vehicle” of the metaphor) and uncertainty (“target” or “topic” of the metaphor) impacted on the participants’ judgments more than the mere activation of the simulation of a state of physical instability. Our data support the idea that metaphors in language and cognition can have a powerful influence on our perception and categorization of the world (Cuccio, 2016).

These findings are also consistent with the Embodied Cognition Hypothesis according to which our conceptual system is strongly tied to our bodily experiences and, specifically, with those studies showing that bodily states of instability/stability influence people’s evaluative judgments and opinions in different and opposite ways (Kille et al., 2013; Landau et al., 2010; Williams, 2024): as already mentioned, in line with these previous studies, our findings seem to show that metaphorical instability expressed in language parallels the effects seen in physical instability on people attitudes. Moreover, these data indirectly corroborate and further extend findings on the embodiment of persuasion which have largely shown that bodily responses (e.g., heartbeat), postures (e.g., standing), and movements (e.g., approach or avoidance) influence persuasion [for a review, Petty and Brinol, 2008; Landau, 2016], confirming the inextricable link between bodily experiences, language and beliefs. Our data seem to show that even a linguistic representation of the body influences persuasion. In addition to this, these data force us to reflect on the role of bodily experience in language and cognition. Results from this study seem to suggest that the impact of the bodily component in language and cognition is greater when the body is conceptualized as part of a metaphorical construction. Indeed, whereas we can presume that in all the three conditions (stable metaphors, unstable metaphors, unstable literal sentences) a simulation of a bodily state takes place, it must be stressed that the effect of the body in persuasion could be greater when the linguistic representation of the body is part of a metaphorical construction, as it is shown from the comparison between unstable metaphors and unstable literal sentences. This might be due to the specific characteristics of metaphor processing that likely leads to a stronger and longer simulation of the vehicle or source domain of the metaphor compared to the simulation which takes place during the comprehension of literal expressions (Cuccio et al., 2014; Cuccio, 2022). Pragmatic processes might be more strongly involved during the construction of metaphorical meaning, leading to a longer activation of the source domain of the metaphor. In addition to this, which might explain why the linguistic representation of a bodily state has a greater impact on persuasion when it is part. Unstable metaphors, in other words, prompted people to make inferences that led them to consider the current and future situation as highly precarious. The projection of inferences from the domain of physical instability to the domain of life conditions was driven by metaphorically structured cognitive representations (Thibodeau et al., 2019).

Overall, our study could have important implications for the understanding of how metaphorical language can shape our bodily experiences and attitudes, suggesting that the use of certain metaphors can have unintended consequences and that we should be careful about which metaphors we use in communication (Carapezza, 2021; Ervas et al., 2021).

In sum, our study provides further evidence of the complex interplay between language, bodily experiences and attitudes, and highlights the need for more research in this area. Future research could further investigate the underlying mechanisms that link metaphorical language, bodily experiences, and attitudes. For example, it would be interesting to investigate whether these effects are mediated by changes in physiological arousal or emotional state.

## Data Availability

The datasets presented in this study can be found in online repositories. The names of the repository/repositories and accession number(s) can be found at: “Unstable metaphors, uncertain minds: how metaphors shape judgements and opinions”: https://osf.io/8wgae/?view_only=7ce40895aac44b86a978509ce8a54984.
